# Optimal Skin Incision for the Surgical Treatment of De Quervain Tenosynovitis: A Systematic Review and Meta-Analysis

**DOI:** 10.3390/medicina62030590

**Published:** 2026-03-20

**Authors:** Dimitrios Kitridis, Eleni Karagergou, Alexandros Givissis, Konstantinos Tsikopoulos, Leonidas Pavlidis, Michael Potoupnis, Panagiotis Givissis

**Affiliations:** 1 Faculty of Health Science, School of Medicine, 1st Orthopaedic Department, Aristotle University of Thessaloniki, 54124 Thessaloniki, Greece; 2 1st Orthopaedic Department, 424 Army General Training Hospital, 56429 Thessaloniki, Greece; 3 School of Medicine, European University of Cyprus, Nicosia 1016, Cyprus; 4 Faculty of Health Science, School of Medicine, 2nd Orthopaedic Department, National and Kapodistrian University of Athens, 11527 Athens, Greece; 5 Orthopaedic Department, North Bristol NHS Trust, Bristol BS10 5NB, UK; 6 Faculty of Health Science, School of Medicine, Department of Plastic Surgery, Aristotle University of Thessaloniki, 54124 Thessaloniki, Greece; 7 Faculty of Health Science, School of Medicine, 3rd Orthopaedic Department, Aristotle University of Thessaloniki, 54124 Thessaloniki, Greece

**Keywords:** de Quervain’s tenosynovitis, longitudinal incision, transverse incision, first dorsal compartment release, surgical treatment, systematic review, meta-analysis

## Abstract

*Background and Objectives:* De Quervain tenosynovitis (DQT) is a stenosing overuse condition of the synovial sheath of the first extensor compartment of the wrist. Open surgical release of the first dorsal compartment remains a standard intervention for DQT when conservative treatments fail. This systematic review evaluated the comparative efficacy of transverse versus longitudinal skin incisions for open release of the first dorsal compartment in DQT. *Materials and Methods:* Four studies with 259 patients were included in the review. Data from 17 patients were unavailable due to loss to follow-up; therefore, 243 wrists (242 patients) were included in the quantitative analysis. The transverse incision group consisted of 114 cases, and the longitudinal incision group of 129 cases. The primary outcome of the review was the incision-related incidence of injuries to adjacent anatomical structures, including injuries to the superficial branch of the radial nerve (SBRN), tendon injuries, and vein injuries. Secondary outcomes included hypertrophic scar formation, wound infection, and postoperative changes in pain severity measured using a visual analog scale (VAS). *Results:* Although there was a lower rate of SBRN injury in the longitudinal group (5.4% vs. 7% in the transverse group), the difference did not meet statistical significance (OR = 2.17; 95% CI, 0.39–11.99; *p* = 0.37; *I*^2^ = 30%). Similarly, there was no significant difference in the risk of vein injury (RD = 0.06; 95% CI, −0.03 to 0.14; *p* = 0.21; *I*^2^ = 61%), hypertrophic scar formation (OR = 1.39; 95% CI, 0.32 to 6.04; *p* = 0.66; *I*^2^ = 35%), and wound infection (RD = 0.00; 95% CI, −0.03 to 0.03; *p* = 0.93; *I*^2^ = 0%). Although both approaches resulted in significant pain improvement, no statistically significant difference in postoperative pain was observed between incision types, as assessed by the VAS for pain (mean difference = 0.30; 95% CI, −0.70 to 1.30; *p* = 0.56; *I*^2^ = 43%). *Conclusions:* No significant differences were identified between incision techniques for DQT in terms of complication rates and postoperative pain outcomes. However, the available evidence is limited, and future high-quality trials are necessary to determine any clinically meaningful difference. Therefore, incision selection should be individualized based on surgeon preference, patient-specific anatomy, and procedural complexity. Despite the technique used, meticulous surgical technique is essential to prevent postoperative complications.

## 1. Introduction

De Quervain tenosynovitis (DQT) is a common musculoskeletal disorder of the wrist, characterized by pain, tenderness, and swelling along the radial aspect of the wrist, localized over the first dorsal extensor compartment. The condition arises from inflammation and thickening of the synovial sheath surrounding the first dorsal extensor compartment tendons (the abductor pollicis longus [APL] and extensor pollicis brevis [EPB]) and their synovial sheath [1,2]. Inflammation and narrowing of the fibro-osseous tunnel that contains these tendons can lead to mechanical irritation, restricted tendon gliding, and progressive pain during thumb and wrist motion. This condition is frequently associated with repetitive or forceful activities involving wrist deviation and thumb movement. As a result, it affects individuals performing repetitive hand use (including manual labor, typing, sports, and household tasks), resulting in substantial functional impairment, reduced grip strength, and reduced quality of life [3].

The initial management of DQT is usually conservative. Standard non-operative treatment options include activity modification, wrist and thumb immobilization with a splint, non-steroidal anti-inflammatory drugs, and corticosteroid injections [2,4,5]. While most patients initially respond to conservative management, surgical intervention is indicated in persistent or severe cases [2,4,5].

The surgical release of the first dorsal compartment is the standard operative treatment for refractory DQT, aiming to decompress the first dorsal compartment and restore normal tendon gliding [6]. Both transverse and longitudinal incisions have been employed in the surgical management [2]. Each technique presents distinct advantages and potential complications. The transverse incision, oriented parallel to Langer’s lines of skin tension, is often preferred for minimized surgical scarring and improved cosmetic outcomes; however, it is associated with an increased risk of iatrogenic injury of the superficial branch of the radial nerve (SBRN) [4,7]. Conversely, the longitudinal incision is frequently considered the safer choice, as it runs parallel to the course of SBRN and facilitates easier identification and protection of the SBRN [8,9]. Furthermore, the longitudinal approach can provide improved exposure of potential variations in compartment anatomy, such as the presence of an intracompartmental septum, or the existence of multiple tendon slips [8,9]. Additionally, this approach is associated with a reduced risk of palmar tendon subluxation due to the more dorsal release of the compartment sheath and can be easily extended proximally or distally if required [8,10].

Despite their theoretical advantages and disadvantages and widespread use, comparative evidence evaluating the safety and effectiveness of these techniques remains limited. Most available studies include relatively small patient cohorts, and their findings have not been comprehensively synthesized. Given these considerations, a systematic synthesis of the existing literature is warranted. This systematic review and meta-analysis aim to integrate the current body of literature to assess the outcomes related to transverse versus longitudinal incisions in the management of DQT. Specifically, we investigated the rates of superficial SBRN lesions, the development of hypertrophic scars, and other intraoperative complications, as well as postoperative outcomes. Through a thorough analysis of the existing evidence, this study aims to improve clinical decision-making and refine surgical techniques to achieve better patient outcomes. By providing a comprehensive evaluation of the benefits and risks associated with each incision type, this study seeks to improve patient outcomes and enhance reproducibility of surgical practice.

## 2. Methods

In the current systematic review, we followed the PRISMA guidelines (Preferred Reporting Items for Systematic Reviews and Meta-Analyses), ensuring transparent reporting of study selection, data extraction, and synthesis (Table S1) [11].

### 2.1. Inclusion and Exclusion Criteria

We included clinical studies comparing outcomes between longitudinal and transverse incision for DQT. Inclusion criteria were:

• Studies including patients with DQT treated surgically after failure of conservative management.
• Studies reporting separate clinical outcomes for transverse and longitudinal incisions for DQT.
• Studies including at least 10 patients to ensure sufficient data for analysis.
• Studies including patients 18 years or older.

Exclusion criteria were:

• Studies not reporting clinical outcomes (i.e., reviews, cadaveric or biomechanical studies, letters to the editor, conference abstracts).
• Studies reporting incomplete or mixed results without distinction between incision types.
• To achieve homogeneity in the treatment arms, studies considering adjuvant procedures (such as procedures for tendon subluxation or procedures for additional pathologies) were also excluded.

### 2.2. Data Sources and Search Process

Two independent reviewers screened abstracts and full-text articles in the Medline, Scopus, and Cochrane Central Register of Controlled Trials (CENTRAL) databases, as well as a manual review of the reference lists of relevant articles up to January 2026. No language or publication date restrictions were applied. Any discrepancies were resolved through consultation with a third reviewer.

For the search strategy, the following search strings were used:

• Medline: (tendin* OR teno* OR tendo* OR bursitis OR parateno*) AND (quervain OR dequervain);
• Scopus: (tendin* OR teno* OR tendo* OR bursitis OR parateno*) AND (quervain OR dequervain);
• Cochrane Central Register of Controlled Trials (CENTRAL): (tendin* OR teno* OR tendo* OR bursitis OR parateno*) in All Text AND (quervain OR dequervain) in All Text.

The asterisk symbol (*) was used as a truncation operator to capture all variations of the root terms.

### 2.3. Data Extraction

Two independent reviewers extracted data from eligible studies. Data extraction included:

• Study characteristics (study design, year of publication, and the country in which each trial was conducted).
• Intervention details (type of incision, surgical technique).
• The number of participants enrolled, the patient demographics, as well as the follow-up completion.
• All outcome measures, follow-up time points and observations.
• All details used for the assessment of risk of bias and the study quality were recorded.

### 2.4. Study Outcomes

The main outcome of the review was the incidence of injuries to adjacent anatomical structures, including injuries to the superficial branch of the radial nerve (SBRN), tendon injuries, and vein injuries. Secondary outcomes included hypertrophic scar formation, wound infection, and postoperative change in pain severity measured in terms of Visual Analog Scale (VAS) [12].

### 2.5. Assessment of Methodological Quality

Two reviewers assessed the methodological quality of the randomized controlled trials (RCTs) using the revised “Cochrane Risk of bias-2” (RoB-2) tool [13]. In the RoB-2 tool, bias is evaluated in five domains: randomization, deviations from intended interventions, missing outcome data, measurement of outcomes, and selection of the reported result. Each domain was judged as low risk, some concerns, or high risk of bias, leading to an overall risk of bias classification.

Non-randomized studies were assessed using the Coleman methodology score [14], which evaluates ten methodological criteria: study size, follow-up adequacy, surgical procedure description, type of study, outcome assessment, diagnostic criteria, description of postoperative rehabilitation, reporting of complications, use of outcome measures, and clarity of inclusion/exclusion criteria. Each criterion is assigned a score, and a score of 100% is considered the optimal study quality score.

To rate the overall quality of evidence of the review, the Grading of Recommendations, Assessment, Development and Evaluations (GRADE) approach was used [15]. GRADE considers study limitations, inconsistency, indirectness, imprecision, and publication bias to classify evidence quality as high, moderate, low, or very low.

### 2.6. Statistical Analysis

A random-effects model was selected a priori for the meta-analysis, to account for potential heterogeneity between studies [16]. Dichotomous outcomes were analyzed using odds ratios (ORs) and 95% confidence intervals (CIs) according to the Mantel-Haenszel method [17]. For studies with zero events in both groups, ORs could not be estimated; therefore, risk difference (RD) was used as the effect measure, as it allows inclusion of double-zero studies and thus preserves all available data. Continuous outcomes were analyzed using the effect size of mean difference and 95% CIs, also calculated according to the Mantel-Haenszel method [17]. Between-study heterogeneity was assessed using Cochran’s Q statistic and quantified with the *I*^2^ statistic (i.e., *I*^2^ = 0–40%: not important heterogeneity; *I*^2^ = 30–60%: moderate heterogeneity; *I*^2^ = 50–90%: substantial heterogeneity; *I*^2^ = 75–100%: considerable heterogeneity) [18]. Forest plots were used to illustrate the effect size in each study, as well as in the overall estimate. All analyses were performed using Review Manager software version 5.3 (Copenhagen: The Nordic Cochrane Centre, The Cochrane Collaboration, 2014).

## 3. Results

### 3.1. Study Selection and Study Characteristics

**Figure 1:**
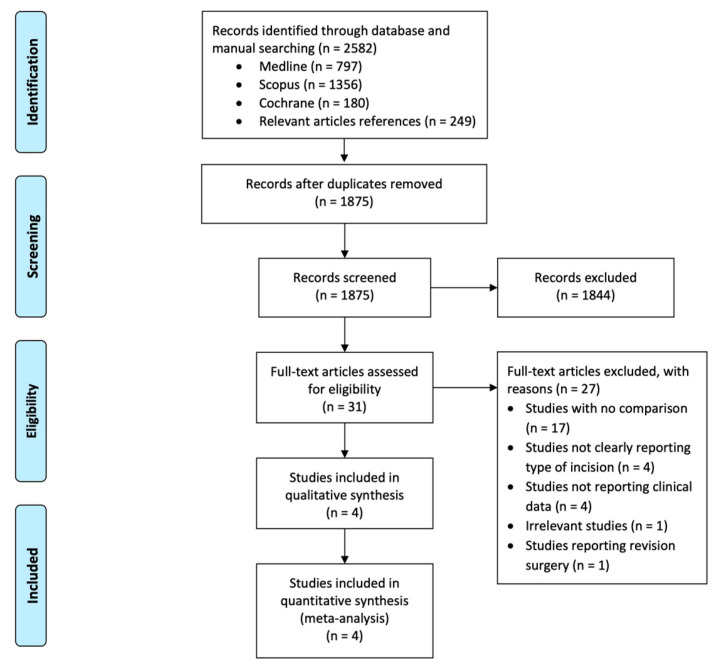
Selection process flow diagram for included studies.

The literature and manual search identified 2059 articles, and after excluding duplicates and irrelevant records, four clinical studies met the inclusion criteria. The trial selection flowchart is presented in Figure 1. Three prospective RCTs [10,19,20] and one retrospective clinical study [21] included 260 cases (259 patients; 41 males and 218 females). Data from 17 patients were unavailable due to loss to follow-up, so 243 wrists (242 patients) were included in the quantitative analysis. The transverse incision group consisted of 114 cases, and the longitudinal incision group of 129 cases.

All source studies were published between 2000 and 2024; three studies were conducted in Asia [10,19,20], and one study in Europe [21]. Key study characteristics, including surgical details and follow-up duration, are summarized in Table 1.

### 3.2. Comparison of Outcomes

In the current systematic review, six outcomes were evaluated comparing the utilization of the transverse versus the longitudinal incisions: SBRN injury, vein injury, hypertrophic scar formation, wound infection, and postoperative pain measured in terms of VAS. No tendon injuries were reported in the trials, suggesting that both incisions preserve tendon integrity. For the remaining outcomes, a random-effects model was used for the analyses.

**Figure 2:**
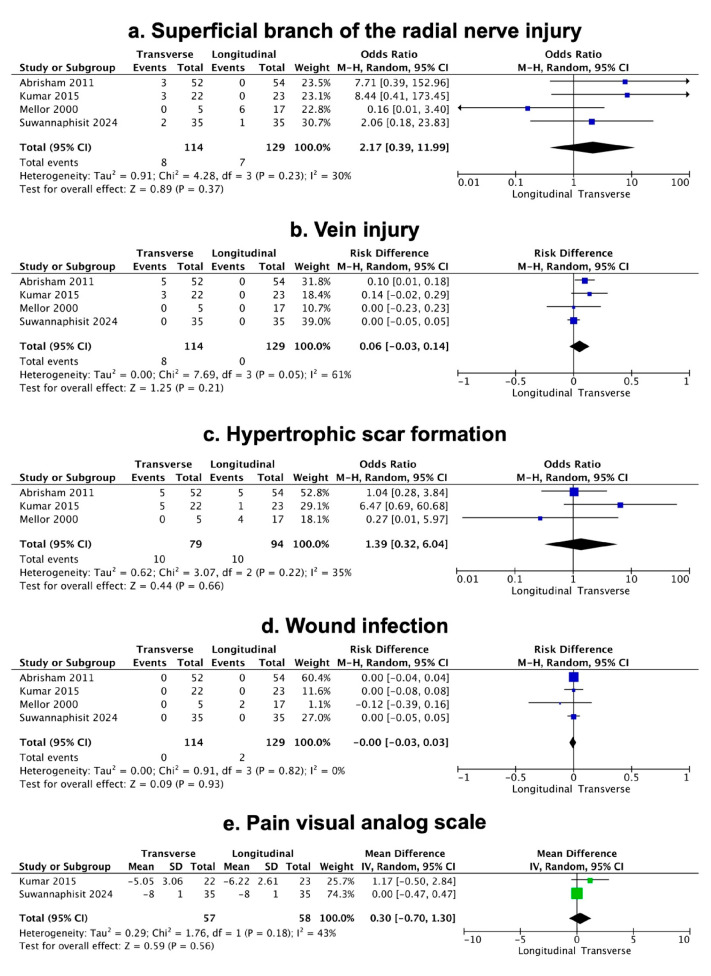
Forest plot demonstrating pooled results. (**a**) Superficial branch of the radial nerve injury; (**b**) vein injury; (**c**) hypertrophic scar formation; (**d**) wound infection; (**e**) pain visual analog scale [10,19,20,21].

SBRN injury was reported in all four included studies. The pooled analysis demonstrated no statistically significant difference in the incidence of SBRN injury between longitudinal and transverse incision groups, with an OR of 2.17; (95% CI, 0.39–11.99; *p* = 0.37) (Figure 2). Although a slightly lower rate of SBRN injury was observed in the longitudinal group (5.4%) compared to the transverse group (7%), the difference did not reach statistical significance.

Similarly, there was no significant difference in the risk of vein injury, with a pooled RD of 0.06 (95% CI, −0.03 to 0.14; *p* = 0.21). All vein injuries occurred in the transverse incision group with a rate of 7% (Figure 2). Four studies contributed data for vein injury.

Three studies evaluated the incidence of hypertrophic scar formation. The pooled analysis showed an OR of 1.39 (95% CI, 0.32 to 6.04; *p* = 0.66) (Figure 2), indicating no statistical difference. Both incision types produced comparable cosmetic outcomes in the included studies.

Regarding wound infection rates, they were very low across studies (0% in the transverse and 1.5% in the longitudinal incision group). Four studies contributed data, with no difference detected between the incision types and a RD of 0.00 (95% CI, −0.03 to 0.03; *p* = 0.93) (Figure 2). This low incidence reflects both the relatively minor nature of the procedure and the adherence to standard perioperative aseptic protocols in the included studies.

Regarding postoperative pain, pooled analysis of two studies demonstrated no significant difference between the two incision types, as assessed by the VAS for pain. The mean difference was 0.30 (95% CI, −0.70 to 1.30; *p* = 0.56) (Figure 2). Both groups experienced substantial improvements in pain, indicating the effectiveness of the surgical release of DQT.

Overall, the meta-analysis of the included studies demonstrated no significant differences between the transverse and longitudinal incisions, suggesting that both approaches have comparable safety profiles and clinical outcomes.

### 3.3. Heterogeneity Analysis

Low heterogeneity was observed for SBRN injury (*I*^2^ = 30%; 95% CI, 0–76%) and hypertrophic scar formation (*I*^2^ = 35%; 95% CI, 0–90%), suggesting consistent findings between studies. Wound infection demonstrated absent heterogeneity (I^2^ = 0%; 95% CI, 0–77%), indicating a consistent low incidence across studies.

Moderate heterogeneity was observed in VAS for pain (*I*^2^ = 43%, 95% CI, 0–94%), likely due to differences follow-up duration and subjective reporting. Vein injury demonstrated moderate-to-substantial heterogeneity (*I*^2^ = 61%; 95% CI, 0–88%), reflecting the small number of events.

### 3.4. Methodological Quality Assessment

**Figure 3:**
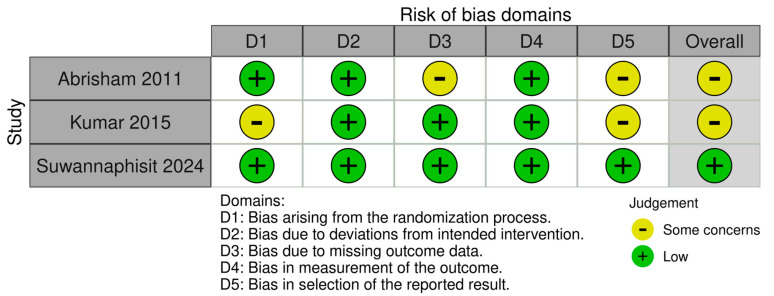
Visual representation of the Risk-of-Bias-2 tool assessment for randomized controlled trials [10,19,20].

The application of the Cochrane RoB-2 tool to the included RCTs revealed varying levels of bias. The studies conducted by Abrisham et al. [10] and Suwannaphisit et al. [19] demonstrated a low risk of bias in random sequence generation and allocation concealment, indicating a robust methodology in participant selection and allocation. In contrast, the study by Kumar [20] raised some concerns regarding the allocation concealment process, which could potentially impact the validity of its findings. Missing follow-up data, results from the study by Abrisham et al. [10] further introduced uncertainty and should be interpreted with some degree of caution. An unclear risk of selection bias was also identified in the studies by Abrisham et al. [10] and Kumar [20] due to the absence of pre-specified trial protocols. Overall, one RCT in our review was classified as low risk [19], while two RCTs [10,20] were categorized within the “some concerns” category of the RoB-2 tool, as shown in Figure 3.

The single non-randomized clinical study [21] was rated using the Coleman Methodology Score and received a 50% score for methodology, indicating a poor methodological quality. Key limitations included the retrospective design, and the small sample size.

Using the GRADE framework, the overall certainty of the evidence was downgraded due to risk of bias (some concerns for two RCTs and high risk of bias for the non-randomized study) and imprecision (small sample sizes, limited number of studies, and wide confidence intervals). No major concerns arose regarding indirectness or inconsistency. Consequently, the overall certainty of evidence for the review was considered low, which reflects the need for further high-quality, adequately powered trials.

## 4. Discussion

In this meta-analysis, no statistically significant differences were observed in the rate of perioperative complications, including injury to the SBRN, vein injury, hypertrophic scar formation, and wound infection, when using either the transverse or the longitudinal incision. However, the confidence intervals for several outcomes were wide, reflecting the limited number of studies and events, and therefore these findings should be interpreted with caution.

To elaborate, injury to the SBRN was observed in eight out of 114 cases in the transverse incision group and in seven out of 129 cases in the longitudinal incision group. Although there was a lower rate of SBRN injury in the longitudinal group (5.4% vs. 7% in the transverse group), the difference did not meet statistical significance (OR = 2.17; 95% CI, 0.39–11.99; *p* = 0.37; I^2^ = 30%). Likewise, the risk of vein injury showed no significant difference (RD = 0.06; 95% CI, −0.03 to 0.14; *p* = 0.21; I^2^ = 61%), although all cases occurred in the transverse incision group (eight out of 114 cases, 7%). Iatrogenic injury of the SBRN, ranging from neurapraxia to complete transection, represents one of the most clinically significant and debilitating complications, causing a painful neuroma with SBRN deficit [21,22]. The crossing superficial branches of the SBRN introduce a problem regarding the choice of the incision in the dorso-radial area of the distal radius [4]. Many incision types have been implemented in clinical practice; the transverse, the longitudinal, the “lazy S” type, and specific-angle incisions, each with its own advantages and disadvantages [4,19]. Many authors have praised the longitudinal incision as the safest for the SBRN, which runs longitudinally along the tendon across the wrist [4,9,10,20,23]. Poublon et al. analyzed the chance of iatrogenic nerve damage in the dorso-radial aspect of the wrist using computer-assisted anatomy mapping to map the course of the nerves in the forearm in a cadaveric study [4]. They reported a safe incision distance of 43.5 ± 18.2 mm between the first two branches of the SBRN when the longitudinal incision is used, and a safe distance of 11.4 (range 0–26.3) mm with the transverse incision, implicating a higher risk of iatrogenic nerve damage with the transverse approach [4]. Although a higher proportion of SBRN injuries was observed in the transverse group, the difference did not reach statistical significance and the confidence interval was wide, reflecting the small number of events.

In the present review, ten cases of hypertrophic scar formation were observed in each group (12.7% for the transverse vs. 10.6% for the longitudinal group), with no significant between-group difference (OR = 1.39; 95% CI, 0.32 to 6.04; *p* = 0.66; I^2^ = 35%). Bruner had investigated the skin incision alternatives for the decompression of the first dorsal compartment in cases of DQT and suggested that the transverse incision results in a less prominent scar because it is placed parallel to the skin creases [7]. In a series of 62 patients treated operatively for DQT, Le Viet and Lantieri reported that the transverse incision provided the best esthetic result [24]. Altay et al. also found cosmetically acceptable scars in all 34 patients treated with transverse incision [25]. Conversely, Scheller utilized the longitudinal incision in a series of 94 patients and found no hypertrophic scar formation [26]. In the same way, Gundes and Tosun reported satisfactory scars using the longitudinal incision and concluded that the results are cosmetically acceptable for the treatment of DQT [9].

Both incision types resulted in postoperative improvement of the VAS for pain, with no significant difference between the transverse and the longitudinal incision (mean difference = 0.30; 95% CI, −0.70 to 1.30; *p* = 0.56; I^2^ = 43%). Kumar et al. reported postoperative pain relief for both the transverse and the longitudinal incision groups (mean VAS difference −5.05 ± 3.06 for the transverse group and −6.22 ± 2.61 for the longitudinal group) and found no between-group difference [20]. In the trial by Suwannaphisit et al., the longitudinal incision group presented with a notable and statistically significant reduction in pain VAS at 2 and 6 weeks compared to the transverse incision group, although at the 12-week mark, no significant difference in pain relief was observed between the two groups (mean VAS difference −8 ± 1 for both groups) [19]. Regarding the effectiveness of the surgical treatment in terms of pain relief, the results of the current review agree with the recent literature, in which surgical release for DQT is beneficial in patients with persistent symptoms, with full resolution of symptoms in up to 95% of patients [27], and a satisfaction rate of 97.5% [28]. This substantial postoperative pain improvement supports the efficacy of the surgical release for DQT, irrespective of incision orientation.

The inadequate decompression of the compartment sheath is an important complication, which may occur due to diminished visualization of the surgical field and the compartment variations, like the presence of a septum [26]. To avoid inadequate release, the ideal surgical approach to the first dorsal compartment should provide good exposure of the entire radial aspect of the wrist [9]. The incision should also be easily extendable if further exploration of the structures is deemed necessary. Several authors advocate the longitudinal incision for better visualization of the structures in the operating field and the ease of diagnosing compartment variations [9,10,20]. A disadvantage of the transverse incision is that it may provide limited exposure to the surgical area, thus complicating the procedure [19]. The current review could not compare the incision types regarding the recurrence of the symptoms or inadequate release of the first dorsal compartment, as it was not reported in the included studies.

The available evidence indicates that both incision types are associated with postoperative pain improvement, with no statistically significant differences identified in complication rates across the included studies [2,23]. Nevertheless, the estimates of our meta-nalysis were characterized by relatively wide confidence intervals, indicating imprecision, and the available data may be insufficiently powered to detect differences in infrequent complications. We found a trend of lower rates of SBRN damage in the longitudinal incision group, although the difference was not statistically significant. This trend aligns with the anatomical considerations reported by Poublon et al., that the longitudinal incision offers wide exposure and less chance of iatrogenic nerve damage [4]. Regardless of the incision used, careful blunt dissection, protection of the SBRN branches, and direct visualization of the first dorsal compartment are essential for minimizing complications.

### 4.1. Limitations

Our study was subject to a number of limitations that warrant cautious interpretation. First, the inclusion of a retrospective study [21] may introduce data collection or patient selection bias to the results. However, the inclusion of this study in the analysis was necessary to increase the sample size and improve the statistical power of the review, due to the limited number of comparative studies for inclusion. Second, the included studies did not report uniform functional outcomes scores. Methodologically, the small number of included studies (two to four per outcome) and inconsistent covariate reporting prevented the meta-regression and subgroup analyses to explore heterogeneity. Heterogeneity across studies was moderate for pain VAS and moderate-to-substantial for vein injury. This may be explained by the relatively small number of included studies, the small number of events, and the subjective reporting of pain. To account for heterogeneity, a random-effects model was used for the analyses, as it incorporates both within-study and between-study variance. Notably, heterogeneity estimates were also imprecise: 95% confidence intervals for I^2^ were wide and included 0% for all outcomes, reflecting the small number of studies per outcome and limiting the reliability of heterogeneity assessments. All these limitations reflect the need for future large-scale, high-quality trials using standardized, validated instruments.

Another important limitation relates to the statistical precision of the pooled results. The small number of studies and relatively few events resulted in wide confidence intervals and limited statistical power, particularly for rare complications. Therefore, the absence of statistically significant differences should not be interpreted as definitive equivalence between the incision techniques but rather as an indication that current evidence is insufficient to demonstrate a clear difference.

Finally, the review protocol was not prospectively registered, which may represent a potential source of bias. Nevertheless, the review was conducted following established methodological standards for systematic reviews and predefined eligibility criteria.

### 4.2. Implications for Future Research

Future studies with larger patient populations and prospective designs comparing incision techniques for the surgical treatment of DQT would help provide more precise estimates of complication rates. In particular, rare complications such as SBRN injury were reported in small numbers in the available studies, limiting the ability to draw robust conclusions. More consistent reporting of complications, functional outcomes, and follow-up duration would also improve comparability across studies and provide more solid evidence synthesis in future meta-analyses. Such studies will enable clearer differentiation between incision techniques and provide more definitive guidance for clinical decision-making.

## 5. Conclusions

This meta-analysis did not identify statistically significant differences between longitudinal and transverse incisions for the surgical treatment of DQT in terms of complication rates and postoperative pain outcomes. However, the available evidence is limited by the small number of studies and the low frequency of several complications, and larger, high-quality trials are necessary to determine any clinically meaningful difference. Therefore, the skin incision selection should be individualized based on surgeon experience, procedural complexity, and the patient’s anatomical features. Despite the technique used, meticulous surgical technique remains essential to prevent postoperative complications.

## Figures and Tables

**Table 1:** Characteristics of the included studies.

Study	Type of Study	Number of Patients Enrolled (Males/Females)		Losses to Follow-Up	Age, Years		Anesthesia Type	Tourniquet Use	Follow-Up
		Transverse	Longitudinal		Transverse	Longitudinal			
Abrisham et al. [10], 2011	RCT	60 (12/48)	60 (12/48)	14	46.5	44	General	Yes	3 months
Kumar [20], 2015	RCT	24 (4/20)	24 (2/22)	3	36.3 (SD 6.6)	37.9 (SD 7.4)	Brachial plexus block	Yes	6 months
Mellor et al. [21], 2000	Retrospective clinical study	21 (4/17)—1 bilateral		None	45 (range 30–78)		14 local, 8 general	Yes	34 (4–78) months
Suwannaphisit et al. [19], 2024	RCT	35 (4/31)	35 (3/32)	None	64	63	Local	Yes	2, 6, 12 weeks

RCT: randomized controlled trial; SD: standard deviation.

## Supplementary Materials

The following supporting information can be downloaded at: https://www.mdpi.com/article/10.3390/medicina62030590/s1, Table S1: PRISMA 2020 Checklist.
